# FOS Inhibits the Differentiation of Intramuscular Adipocytes in Goats

**DOI:** 10.3390/genes14112088

**Published:** 2023-11-17

**Authors:** Tingting Hu, Zhibin Li, Chengsi Gong, Yan Xiong, Shiyu Sun, Jiani Xing, Yanyan Li, Ruiwen Li, Youli Wang, Yong Wang, Yaqiu Lin

**Affiliations:** 1 College of Animal Science and Veterinary, Southwest Minzu University, Chengdu 610041, China; hutingting232022@126.com (T.H.); 15081462885@163.com (Z.L.); 13608047713@163.com (C.G.); xiongyan0910@126.com (Y.X.); sunshiyu0311@163.com (S.S.); 80300244@swun.edu.cn (J.X.); liyanyan@swun.edu.cn (Y.L.); wangylwy@163.com (Y.W.); wangyong010101@hotmail.com (Y.W.); 2 Key Laboratory of Qinghai-Tibetan Plateau Animal Genetic Resource Reservation and Utilization, Ministry of Education, Southwest Minzu University, Chengdu 610041, China; 3 Key Laboratory of Sichuan Province for Qinghai-Tibetan Plateau Animal Genetic Resource Reservation and Exploitation, Southwest Minzu University, Chengdu 610041, China; 4 Chengdu Women’s and Children’s Central Hospital, School of Medicine, University of Electronic Science and Technology of China, Chengdu 611731, China; liruiwen0001@163.com

**Keywords:** goat, FOS, intramuscular adipocytes, cell differentiation

## Abstract

Goat intramuscular fat (IMF) deposition is precisely regulated by many key genes as well as transcription factors. Nevertheless, the potential of the regulators of goat IMF deposition remains undefined. In this work, we reported that the transcription factor FOS is expressed at a low level at the early differentiation stage and at a high level in late differentiation. The overexpression of FOS inhibited intramuscular adipocyte lipid accumulation and significantly downregulated the expressions of *PPARγ*, *C/EBPβ*, *C/EBPα*, *AP2*, *SREBP1*, *FASN*, *ACC*, *HSL*, and *ATGL*. Consistently, the knockdown of FOS, facilitated by two distinct siRNAs, significantly promoted intramuscular adipocyte lipid accumulation. Moreover, our analysis revealed multiple potential binding sites for FOS on the promoters of *PPARγ*, *C/EBPβ*, and *C/EBPα*. The expression changes in *PPARγ*, *C/EBPβ*, and *C/EBPα* during intramuscular adipogenesis were opposite to that of FOS. In summary, FOS inhibits intramuscular lipogenesis in goats and potentially negatively regulates the expressions of *PPARγ*, *C/EBPβ*, and *C/EBPα* genes. Our research will provide valuable data for the underlying molecular mechanism of the FOS regulation network of intramuscular lipogenesis.

## 1. Introduction

Owing to rising consumer awareness of meat-associated quality, consumer demand for high-quality goat meat has increased significantly [1]. Meat quality is influenced by various factors, in which the IMF content plays a critical role in determining meat-quality traits [2]. An appropriate IMF content can improve the tenderness, juiciness, and flavor of meat products [3]. Animal IMF deposition results from a combination of adipocyte proliferation and hypertrophy. Adipocyte differentiation is a critical pathway for animal IMF accumulation, which is tightly controlled by numerous important genes and transcription factors [4]. Therefore, it is very important to unravel the molecular mechanism by which key transcription factors regulate the differentiation of adipocytes in goats.

The transcription factor FOS is a nuclear-like protein with a basic leucine zipper encoded by the proto-oncogene *c-fos*. FOS is a transcription factor superfamily, including FOS, v-FOS, FOSB, Fra1, and Fra2, which together with members of the proto-oncogene JUN family (c-Jun, JunB, and JunD) and the activating transcription factor (ATF) protein family constitute the AP-1 transcription factor complex [5,6]. FOS is involved in a variety of biogenic processes in vivo and is closely linked to the differentiation of chondrocytes and osteoclasts, as well as tumor formation and carcinogenesis [7,8,9,10]. It has been reported that FOS regulates the growth and differentiation of adipocytes [11]. For example, the knockdown of the *FOS* gene reduced lipid droplet accumulation and inhibited the differentiation of 3T3-L1 adipocytes [12]. Meanwhile, another study showed that all-trans retinoic acid (atRA) stimulated the interaction of retinoic acid receptor γ (RARγ) with FOS protein, hindering the binding of FOS to PPARγ2, which resulted in a reduction in the expression of PPARγ2 and, ultimately, hindering the differentiation of 3T3-L1 adipocytes [13]. Congenital generalized lipodystrophy (CGL) refers to a syndrome of adipocyte dysplasia caused by mutations in the *FOS* gene promoter in CGL patients, resulting in a reduced expression of FOS that interferes with preadipocyte differentiation [14]. Therefore, considering the variations between species, the effect of FOS on adipogenesis in goat intramuscular adipocytes should be clarified.

This study aims to investigate the impact of FOS on the regulation of adipogenesis in goat intramuscular preadipocytes. First, the localization of FOS expression was detected in goat intramuscular precursor adipocytes. Subsequently, the expression levels of FOS were detected at various stages of lipogenesis in goat intramuscular adipocytes. Then, the role of FOS in intramuscular preadipocyte differentiation was determined by loss and gain of function using siRNAs and an overexpression vector. In addition, the potential target genes of FOS were predicted and compared with FOS, they exhibited contrasting expression patterns at various stages of lipogenesis in intramuscular adipocytes. Taken together, our study results indicate that FOS negatively regulates goat intramuscular adipogenesis and provides new information about the role of FOS in adipogenesis.

## 2. Materials and Methods

### 2.1. Cell Culture

The animal experimentation study received approval from the Laboratory Animal Ethics Committee at Southwest Minzu University and the Animal Disease Control Center in Sichuan Province, China. Jianzhou Daer goats (*Capra hircus*) (N 3) were purchased from Sichuan Tiandi Goat Biological Engineering Co., Ltd. (Chengdu, China). Goat intramuscular preadipocytes were isolated and cultured in accordance with previously described methods [15,16]. Concisely, Longissimus dorsi muscle samples were collected from seven-day-old Jianzhou Daer goats (N = 3), and the samples were sheared. Intramuscular preadipocytes were isolated by digestion with 2 mg/mL of collagenase type II (Gibco, Thermo, Waltham, MA, USA). Finally, the preadipocytes were cultured in growth medium DMEM/F12 supplemented with 10% FBS and 1% P/S.

### 2.2. Construction of Overexpression Vector, siRNA Synthesis, Cell Transfection

Briefly, the coding region of the goat *FOS* gene was amplified using RT-PCR, and the *Kpn* I and *Hin*d III sites were selected as upstream and downstream cleavage sites, respectively, for primer design (Table 1 shows the sequences of the primers.) and for cloning. The FOS overexpression plasmid was digested with restriction enzymes (TaKaRa, Kusatsu, Shiga, Japan) and ligated with T4 DNA ligase (TaKaRa, Kusatsu, Shiga, Japan). The bacterial solution confirmed as positive by PCR was proliferated, and the plasmid was extracted and identified by restriction endonuclease digestion and sequencing. (Biological Biotechnology Co., Ltd., Chengdu, China). Two individual siRNAs targeted for goat *FOS* and a negative control (NC) siRNA were synthesized by Gene Pharma (Shanghai, China). The sequences of the NC and si-RNAs were as follows:

siRNA1: (5′-GUUCCUUCUAUGCAGATTUCUGCUGCAUAGAAGGAACTT-3′);

siRNA2: (5′-GAGAUUGCCAAUCGCUGATTUCAGCAGAUUGGCAAUCUCTT-3′);

NC: (5′-UUCUCCGAACGUGUCACGUT TACGUGACACGUUCGGAGAATT-3′).

Goat FOS overexpression vector and siRNA transfections were performed using TurboFect (Thermo, Waltham, MA, USA). Intramuscular preadipocytes were transfected with the transfection reagent (Thermo, Waltham, MA, USA) at a 70% confluence. Then, the medium was changed to a medium containing 50 µmol/L of oleic acid, and differentiation was induced for 2 days at 16 h post transfection.

### 2.3. Oil Red O Staining

The cell culture method was the same as that described above. First, goat intramuscular adipocytes were fixed with 4% formaldehyde for 20 min at room temperature. The Oil Red O staining was performed according to the method described by Xiong et al. [15]. Next, Oil Red O dye was added to each well for staining for 15 min. After the stained adipocytes were washed twice with PBS, 200 μL of PBS was added again as the background to observe the shape of the lipid droplets using a microscope (Olympus, Tokyo, Japan). Finally, 1 mL of isopropanol was added to dissolve the Oil Red O dye, and the absorbance value of each well was measured at 490 nm.

### 2.4. Bodipy Staining and DAPI Staining

Bodipy and DAPI stainings were performed according to the method described by Chen et al. [16]. Bodipy and DAPI dyes were first diluted in PBS at a ratio of 1:1000. Then, Bodipy dye was added to each well, and the adipocytes were stained for 10 min away from light and washed again with PBS, and DAPI dye was added for another 10 min. Finally, the aggregation of lipid droplets in adipocytes was observed under a fluorescence microscope and photographed.

### 2.5. Cell RNA Extraction and Quantitative Real-Time PCR

Total RNA was isolated from harvested intramuscular adipocytes, using RNAiso Plus (TaKaRa, Kusatsu, Shiga, Japan). Briefly, goat intramuscular adipocytes were collected and resuspended using RNAiso Plus. Chloroform was added, and the suspension was shaken vigorously and then allowed to stand, followed by centrifugation. The supernatant was then aspirated and transferred to a new tube. Isopropyl alcohol was added to precipitate the RNA, and centrifugation was performed. The liquid above the sediment was discarded, and the precipitate was washed with DEPC ethanol and then centrifuged repeatedly. The supernatant was discarded, and the RNA precipitate was allowed to air-dry. Finally, an appropriate amount of DEPC water was added to dissolve the RNA. The A260/A280 values of the RNA were determined as in the range 1.8–2.0 using a UV spectrophotometer, which met the test requirements. cDNA was reverse-transcribed using a reverse-transcriptase kit (Thermo, Waltham, MA, USA) and the quantitative PCR spiking system described by Chen et al. [16]. The *UXT* gene was used as an internal control. The qPCR primer sequences are listed in Table 1.

### 2.6. Measurement of Cellular Triglyceride Levels

Intramuscular adipocytes were collected after 2 days of differentiation induction and were lysed according to the instructions of the triglyceride determination kit (Applygen, Beijing, China), and the triglyceride content was determined according to the procedures described in the manufacturer’s instructions.

### 2.7. Western Blotting

The total protein from the goat intramuscular adipocytes was extracted using RIPA cell lysate on ice. According to the instructions of the BCA protein assay kit (Biosharp, Shanghai, China), the protein concentration of each sample was determined and denatured at 100 °C. Western blotting analyses were performed as previously described in the literature [17,18]. In brief, each sample was loaded at 30 µg/lane and subjected to 12% SDS-PAGE, and the protein was transferred to a PVDF membrane (Millipore, Billerica, MA, USA). The protein membranes were then incubated with different antibodies at 4 °C. PPARγ, C/EBPβ, and β-actin antibodies (Abcam, Cambridge, UK) were diluted at a ratio of 1:1000. A horseradish peroxidase (HRP)-labeled secondary antibody was diluted at a 1:5000 ratio (Abways, Shanghai, China) and incubated for 1 h at room temperature. Finally, immunodetection was performed using ECL substrate reagents (Bio-Rad, Hercules, CA, USA).

### 2.8. Statistical Analysis

All the data were analyzed for significance using SPSS 26.0 software and expressed as mean ± SEM. The qPCR images were generated using GraphPad Prism 8.0 software. Student’s two-tailed *t*-test was utilized to analyze comparisons between two groups. The one-way ANOVA test was utilized to analyze comparisons between multiple groups, and Duncan’s method was used to demonstrate the significance of the data, where “*” means a significant difference (*p* < 0.05) and “**” means an extremely significant difference (*p* < 0.01).

## 3. Results

### 3.1. Subcellular Localization and Expression Patterns of FOS during Differentiation of Goat Intramuscular Adipocytes

**Figure 1:**
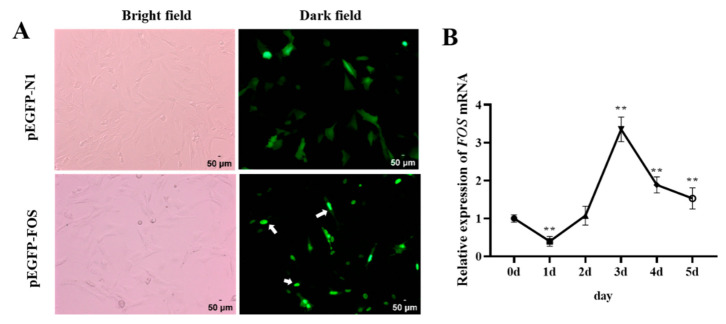
Subcellular localization of FOS and expression patterns during differentiation of goat intramuscular adipocytes. (**A**) The upper panels show the localization of PEGFP-N1, and the bottom panels show the localization of the FOS protein in intramuscular adipocytes, as indicated by the white arrow in the figure. The scale bar represents 50 µm. (**B**) The expression changes in FOS during 0–5 days of induced differentiation of goat intramuscular adipocytes (n = 6). “**” means an extremely significant difference (*P* < 0.01).

Previous research has found that in COS-1 cells, 95% of the FOS proteins are localized in the nucleus, and 5% are in the cytoplasm [19]. To investigate whether the localization of FOS in goat intramuscular adipocytes is consistent with that in COS-1 cells, the FOS overexpression vector (pEGFP-FOS) and pEGFP-N1 were separately transfected in goat intramuscular preadipocytes for 16 h and were observed under an inverted fluorescence microscope. The results showed that the green fluorescence of the pEGFP-N1 vector was uniformly distributed in the whole cell, while the green fluorescence of the FOS protein was mainly distributed in the nucleus (Figure 1A). It was further confirmed that FOS was a nucleoprotein-like protein, and we speculated that the goat FOS protein might mainly play its biological role in the nucleus. Next, the mRNA expression changes in FOS were analyzed during intramuscular adipocyte differentiation. The expression of the FOS gene was low on day 1, reached its highest level on day 3 post-differentiation, and then downregulated after day 4 (Figure 1B). These data suggested that FOS may regulate intramuscular fat production in the late stage of differentiation.

### 3.2. Overexpression of FOS Inhibits Goat Intramuscular Preadipocyte Differentiation

**Figure 2:**
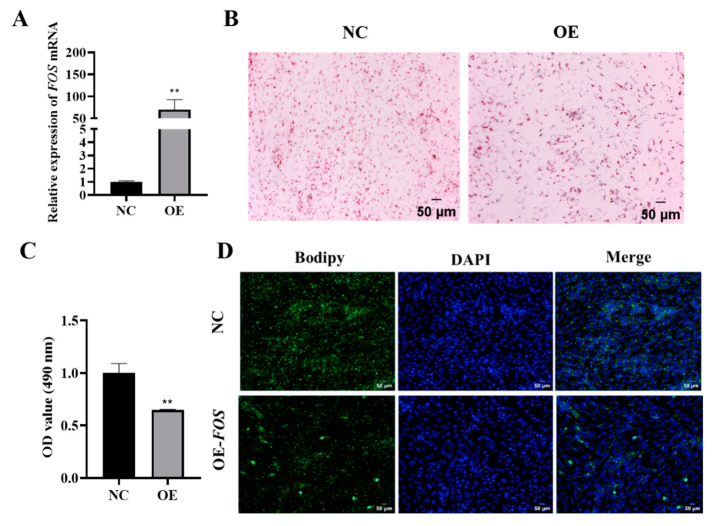
Overexpression of FOS inhibits goat intramuscular adipocyte differentiation. (**A**) Overexpression efficiency of FOS detected using qPCR (n = 6). (**B**,**C**) Oil Red O staining (×200) (n = 3), scale bar represents 50 µm, and the value of OD at 490 nm. (**D**) Bodipy and DAPI stainings (×200); scale bar represents 50 µm. The green fluorescence represents lipid droplets. “**” means an extremely significant difference (*P* < 0.01).

To investigate the mechanism by which FOS regulates lipid production in goat intramuscular adipocytes, a pEGFP-FOS overexpression vector was constructed and transfected in goat intramuscular preadipocytes, and the overexpression efficiency was then detected using qPCR. The qPCR analysis showed a significant 70-fold increase (*p* < 0.01) in the expression of FOS compared to that of the NC (Figure 2A). Furthermore, the Oil Red O staining and extraction assay demonstrated that the overexpression of FOS hindered lipid droplet aggregation in goat intramuscular adipocytes (Figure 2B,C). The Bodipy staining results also showed the same trend. (Figure 2D). These data indicate that the overexpression of FOS inhibited intramuscular fat deposition in goats.

**Figure 3:**
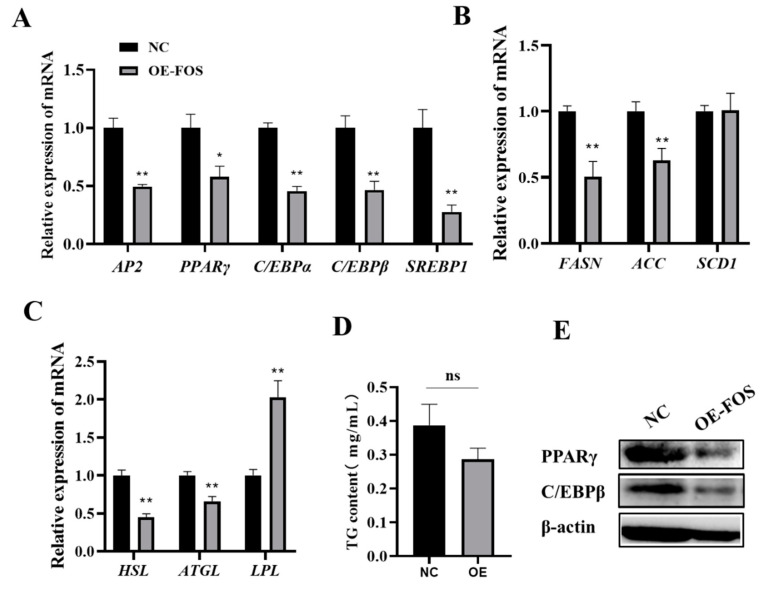
Overexpression of FOS suppresses the expression of adipogenesis genes. (**A**–**C**) The expressions of AP2, *PPARγ*, *C/EBPβ*, *C/EBPα*, *SREBP1*, *FASN*, *ACC*, *SCD1*, *HSL*, *ATGL*, and *LPL* in goat intramuscular adipocytes in NC and OE groups. (**D**) The cellular TG levels in goat intramuscular adipocytes in negative control (NC) and overexpression of FOS treatment groups. (**E**) Protein expressions of PPARγ and C/EBPβ. “*” means a significant difference (*P* < 0.05), “**” means an extremely significant difference (*P* < 0.01) and “ns” means no difference.

Adipocyte differentiation is a major pathway of intramuscular fat deposition in animals and is precisely regulated by a series of key genes and transcription factors [20,21]. To further explore the possible mechanism of the inhibition of lipid droplet aggregation in goat intramuscular adipocytes by the expression of FOS, the expressions of lipogenesis-related genes were also detected. The mRNA levels of *AP2*, *PPARγ*, *C/EBPβ*, *C/EBPα*, and *SREBP1* were significantly decreased in the FOS overexpression group compared to those of the NC (*p* < 0.05) (Figure 3A). In addition, the genes related to lipid metabolism, such as *FASN*, *ACC*, *HSL*, and *ATGL*, were highly significantly downregulated, and *LPL* was highly significantly upregulated (*p* < 0.01) (Figure 3B,C). However, the cellular TG content was not different in the NC and FOS overexpression group (Figure 3D). PPARγ and C/EBPβ protein expressions were inhibited in the FOS overexpression group (Figure 3E). In summary, the overexpression of FOS inhibits the expression of genes related to adipogenesis.

### 3.3. Knockdown of FOS Promotes Goat Intramuscular Preadipocyte Differentiation

**Figure 4:**
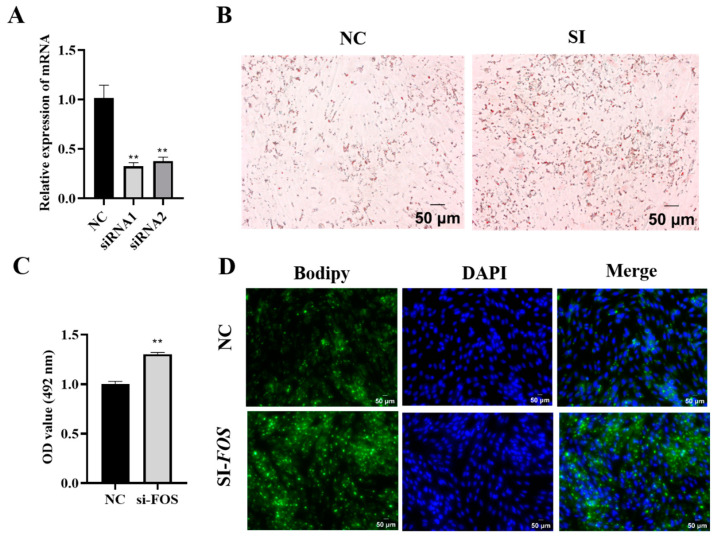
Knockdown of FOS increases accumulation of lipid droplets in goat intramuscular adipocytes. (**A**) The knockdown efficiency of the FOS detection. (**B**,**C**) Oil Red O staining (×200), scale bar represents 50 µm, and the value of OD at 490 nm. (**D**) Bodipy and DAPI stainings (×200); scale bar represents 50 µm. The green fluorescence represents lipid droplets. “**” means an extremely significant difference (*P* < 0.01).

Next, the knockdown of the FOS was achieved using siRNA-mediated techniques in the intramuscular adipocytes of goats. The results showed that the interference efficiency of siRNA1 was 48% and that of siRNA2 was 40% (Figure 4A) compared to those of the NC group. Oil Red O staining and its extraction revealed that the knockdown of FOS significantly promoted lipid droplet aggregation in goat intramuscular adipocytes. (Figure 4B,C). The Bodipy staining results showed the same trend (Figure 4D). In short, the above data suggested that reduced FOS expression increased lipid accumulation.

**Figure 5:**
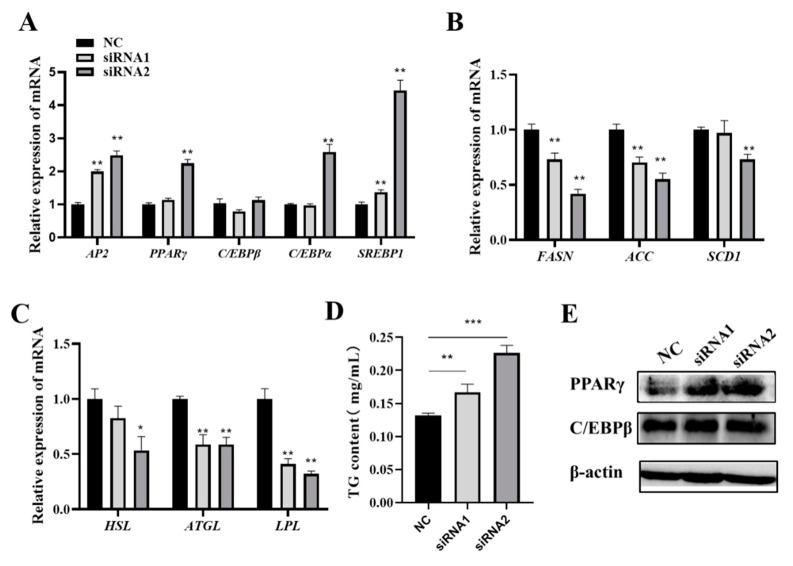
Knockdown of FOS upregulates positive adipocyte differentiation genes and downregulates lipid metabolism-related genes. (**A**–**C**) The expressions of lipogenic-related genes in the NC and knockdown FOS groups. (**D**) Detection of cellular TG contents in the NC and FOS knockdown groups. (**E**) PPARγ and C/EBPβ protein expression levels in NC and knockdown *FOS* groups. “*” means a significant difference (*P* < 0.05), “**” means an extremely significant difference (*P* < 0.01) and “***” means an extremely significant difference (*P* < 0.001).

The expression of lipogenic-related genes was examined after knocking down FOS in adipocytes. The mRNA levels of *AP2*, *PPARγ*, C*/EBPα*, and *SREBP1* were significantly increased in the knockdown groups (Figure 5A). Surprisingly, the expressions of lipogenic and lipolysis genes, including *FASN*, *ACC*, *SCD1*, *HSL*, *ATGL*, and *LPL*, were significantly suppressed (*p* < 0.05) (Figure 5B,C), but the TG content was significantly increased in the knockdown groups (Figure 5D). Meanwhile, PPARγ and C/EBPβ expressions were increased in the knockdown groups (Figure 5E). These results suggested that the loss of function of FOS promoted the expression of intramuscular adipocyte differentiation genes.

### 3.4. FOS Affects Intramuscular Preadipocyte Differentiation by Targeting PPARγ, C/EBPβ, and C/EBPα

**Figure 6:**
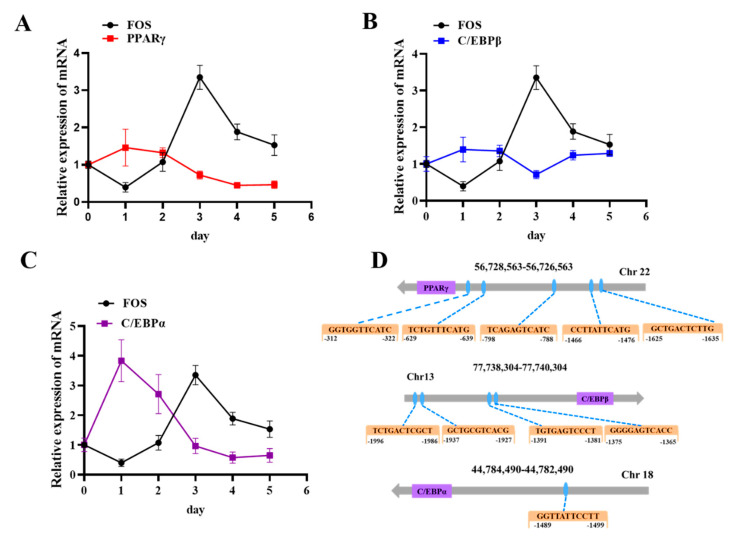
FOS exhibits an expression pattern opposite to those of PPARγ, C/EBPβ, and C/EBPα during adipocyte differentiation. (**A**) the PPARγ expression trend during intramuscular adipogenesis (n = 6). (**B**) The C/EBPβ expression trend during intramuscular adipogenesis. (**C**) The C/EBPα expression trend during intramuscular adipogenesis. (**D**) FOS binding sites on the promoters of PPARγ and C/EBPβ. Blue circles represent the FOS binding sites.

As FOS was found to be a negative regulator of goat preadipocyte differentiation and to repress the expression of adipogenic genes, we hypothesized that FOS may inhibit lipid deposition by targeting downstream genes. According to the relevant literature, FOS can bind to PPARγ and C/EBP promoter sequences and regulate their expressions. Consequently, the expression changes in PPARγ, C/EBPβ, and C/EBPα were examined in adipocytes induced to differentiate from day 0 to day 5. The results showed that the expressions of PPARγ, C/EBPβ, and C/EBPα were opposite to that of FOS in adipocytes induced to differentiate (Figure 6A–C). Then, the transcriptional binding DNA motif of FOS was analyzed using JASPARsoftware (https://jaspar.elixir.no/ 27 September 2023), and the promoter sequences, including PPARγ, C/EBPβ, and C/EBPα, were downloaded from the NCBI website (https://www.ncbi.nlm.nih.gov/ 27 September 2023). As shown in Figure 6D, PPARγ, C/EBPβ, and C/EBPα were predicted as potential targets of FOS, with multiple potential binding sites in their respective promoter regions. All together, these data suggested that FOS may influence intramuscular adiposity by regulating the expressions of PPARγ, C/EBPβ, and C/EBPα.

## 4. Discussion

Intramuscular fat deposition is precisely regulated by numerous key genes and transcription factors. Therefore, it is important to reveal the transcription factors that influence adipogenesis to enhance intramuscular fat deposition. Herein, we presented evidence that FOS serves as a negative regulator of intramuscular adipocyte differentiation. Our research provides important theoretical support for refining the molecular network of the key transcription factors regulating intramuscular fat deposition in goats. Previous studies have reported that FOS is encoded by the proto-oncogene c-fos, a nuclear-like protein with an alkaline zipper. In COS-1 cells, 95% of the FOS proteins are localized in the nucleus, and 5% are in the cytoplasm [19]. In this study, we have demonstrated that FOS is localized in the nucleus in goat intramuscular adipocytes and is a nuclear-like protein. We hypothesized that goat FOS proteins may play their biological roles mainly in the nucleus. Barutcu found that the sustained expression of FOS inhibits the terminal differentiation of primary muscle progenitor cells [22]. To investigate the expression pattern of FOS during the differentiation of goat intramuscular adipocytes, the changes in FOS expression in intramuscular adipocytes were examined from day 0 to day 5 of differentiation, and the results revealed that FOS is lowly expressed in the pre-differentiation stage and highly expressed in the late differentiation stage. As mentioned above, we speculated that FOS may play a role in the late stage of differentiation, and the following results suggested that FOS is a negative regulator of genes related to intramuscular lipid droplet aggregation and lipogenesis in goats. First, the overexpression of FOS reduced the intramuscular adipocyte lipid content, as confirmed by the Oil Red O and Bodipy staining results. Moreover, the overexpression of FOS inhibited the expressions of intramuscular adipogenic genes, such as *PPARγ*, which is a major transcription factor in adipogenesis and has two isozymes, PPARγ1 and PPARγ2 [23]. Previous studies have demonstrated that γ1 was highly expressed in adipose, liver, spleen, and heart tissues, whereas γ2 was highly expressed in adipose tissue; yet, lower expression levels of γ1 and γ2 were found in skeletal muscle tissue [24]. The overexpression of PPARγ2 stimulated intramuscular preadipocyte differentiation in pigs and promoted the expression of lipogenesis-related genes [25]. In addition to PPARγ, the C/EBP family is the major regulator of adipocyte differentiation [26]. The C/EBP transcription factor family includes six members; currently, the most influential on adipocyte differentiation are C/EBPα, β, and δ [27]. It has been shown that PPARγ and C/EBPα are involved in regulating late adipocyte differentiation and control the expressions of downstream genes, such as aP2 and LPL [4]. C/EBPβ is highly expressed in early adipocyte differentiation, and its expression level is gradually downregulated in late adipocyte differentiation [28]. Taken together, PPARγ, C/EBPα, and C/EBPβ are essential factors for adipocyte differentiation. Our results demonstrated that overexpression of FOS inhibits the expression of C/EBPα and C/EBPβ, which is one of the reasons for impaired adipocyte differentiation. *LPL* is highly expressed in adipose tissue, muscle, and liver [29], and catalyzes the hydrolysis of triglycerides to glycerol and free fatty acids [30]. Overexpression of LPL attenuates lipid droplet accumulation and improves glucose metabolism in the liver of HFD-fed mice [31]. Our results showed that *LPL* was upregulated in FOS-overexpressing cells and was downregulated in the knockdown cells. We hypothesized that the main reason for the inhibition of lipid accumulation by the overexpression of FOS was a decrease in triglyceride synthesis, which was also supported by a decrease in the mRNA level of LPL in siRNA-treated cells. What is surprising is that the lipid metabolism-related genes, *FASN*, *ACC*, *HSL*, and *ATGL*, were significantly downregulated in overexpression and knockdown cells. FASN can produce long-chain fatty acids in the presence of malonyl coenzyme A, whereas ACC is the rate-limiting enzyme for the biosynthesis of long-chain fatty acids, and both are key enzymes in lipogenesis [32,33]. ATGL catalyzes the conversion from triglycerides to diacylglycerols and fatty acids, and the knockout of ATGL in vascular stromal cells and fibroblasts resulted in a decrease in TG hydrolase activity and an increase in TG content [34]. HSL is also a lipolytic enzyme, and adipocyte-specific HSL-knockout mice exhibit excessive hepatic fat accumulation and diminished lipolytic activity after being fed a high-fat diet [35]. The extent of fat deposition depends on the balance between TG synthesis and catabolism [36]. When the ability to synthesize triglycerides decreases faster than the rate of catabolism, this leads to a decrease in triglyceride levels and, conversely, an increase. This is one of the main reasons that the overexpression of FOS inhibited lipid accumulation, whereas the knockdown of FOS promoted lipid accumulation. It has been reported that FOS proteins are highly expressed in NASH mice, and microRNA29c can regulate the course of NASH by targeting FOS proteins [37]. The specific binding from SPARC to C-FOS leads to a decrease in AP-1 activity, which inhibits adipogenesis but promotes osteoclast differentiation [38]. These previous findings are inconsistent with the results of this study, and we speculated that the various roles played by FOS in adipogenesis may be attributed to species variations and inconsistent mechanisms of action.

Bioinformatics analysis has demonstrated that *PPARγ*, *C/EBPβ*, and *C/EBPα* promoters possess multiple binding sites for FOS. Moreover, it has been reported that *PPARγ*, *C/EBPα*, and *C/EBPβ* exhibit promoter-binding activities with FOS [13,39,40,41]. Therefore, we hypothesized that PPARγ, C/EBPα, and C/EBPβ may be potential target genes for the FOS regulation of intramuscular adipocyte differentiation in goats. The experimental results indicated that the mRNA levels of PPARγ, C/EBPβ, and C/EBPα changed during intramuscular adipogenesis and were opposite to that of FOS. Meanwhile, the expression levels of PPARγ and C/EBPα were remarkably upregulated after the knockdown of FOS. It is a discrepancy that the knockdown of *c-Fos* inhibits 3T3-L1 cell differentiation by affecting the PPARγ2 promoter activity [12]. We hypothesized that the inconsistent regulatory effects of FOS on PPARγ may be due to differences in species and cell types. This might also be because FOS is not a single transcription factor but is a part of the AP-1 complex. It has been reported that FOS forms heterodimers with JUN and ATF, which then bind to specific sequences within the regulatory regions of target genes [42,43]. Perhaps, because of differences in the dimer composition in adipocytes, the activation or repression effect on target genes was different [42]. It has been shown that C/EBPα could interact with FOS and strongly induce monocytic differentiation [44]. In addition, the overexpressions of FOS and its family members, Fra-1 and FosB, affect lipogenesis by inhibiting C/EBP activity [45,46]. As mentioned above, we speculated that FOS may influence intramuscular adiposity by regulating the expressions of PPARγ, C/EBPβ, and C/EBPα.

## 5. Conclusions

In conclusion, our findings highlight that FOS serves as an inhibitory factor in the differentiation of intramuscular adipocytes in goats. This inhibition is associated with notable changes in the levels of key adipogenic transcription factors, including *PPARγ*, *C/EBPβ*, and *C/EBPα*, along with alterations in the expressions of critical lipid-metabolism genes, such as *FASN*, *ACC*, and *LPL*. These results not only underscore the potential of FOS as a novel target for improving the quality of goat meat but also provide essential data for further investigation into the intricate molecular mechanisms governing the FOS regulatory network in IMF deposition.

## Figures and Tables

**Table 1:** Primers for quantitative real-time PCR (qPCR).

Gene	Forward Primer (5′-3′)	Reverse Primer (5′-3′)
*FOS*	GCTTCAACGCCGACTACGAG	AAGGAGTCTGCCGGTGAGTG
*AP2*	TGAAGTCACTCCAGATGACAGG	TGACACATTCCAGCACCAGC
*PPARγ*	AAGCGTCAGGGTTCCACTATG	GAACCTGATGGCGTTATGAGAC
*C/EBPβ*	CAAGAAGACGGTGGACAAGC	AACAAGTTCCGCAGGGTG
*C/EBPα*	CCGTGGACAAGAACAGCAAC	AGGCGGTCATTGTCACTGGT
*SREBP1*	AAGTGGTGGGCCTCTCTGA	GCAGGGGTTTCTCGGACT
*FASN*	TGTGCAACTGTGCCCTAG	GTCCTCTGAGCAGCGTGT
*ACC*	GGAGACAAACAGGGACCATT	ATCAGGGACTGCCGAAAC
*SCD1*	TCGTGCCGTGGTATCTATGG	GGGGTTGATGGTCTTGTCGT
*HSL*	AGGGTCATTGCCGACTTCC	GTCTCGTTGCGTTTGTAGTGC
*ATGL*	GGAGCTTATCCAGGCCAATG	TGCGGGCAGATGTCACTCT
*LPL*	GAGGCCTTGGAGATGTGGAC	AATTGCACCGGTACGCCTTA
*UXT*	GCAAGTGGATTTGGGCTGTAAC	ATGGAGTCCTTGGTGAGGTTGT

## Data Availability

Data are contained within the article.

## Abbreviations

IMF	Intramuscular fat
AP-1	Activator protein-1
PPARγ	Peroxisome proliferator-activated receptor γ
C/EBPβ	CCAAT enhancer binding protein β
C/EBPα	CCAAT enhancer binding protein α
AP2	Fatty-acid binding protein
SREBP1	Sterol regulatory element binding protein-1
FASN	Fatty-acid synthase
ACC	Acetyl-coenzyme A carboxylase
SCD1	Stearoyl-coenzyme A desaturase 1
HSL	Hormone-sensitive lipase
ATGL	Adipose triglyceride lipase
LPL	Lipoprotein lipase
UXT	Ubiquitously expressed transcript gene
NC	Negative control
OE	Overexpression
HFD-fed	High-fat-diet feed
NASH	Nonalcoholic steatohepatitis
SPARC	Secreted protein acidic and rich in cysteine
